# A Panel of CA19-9, Ca125, and Ca15-3 as the Enhanced Test for the Differential Diagnosis of the Pancreatic Lesion

**DOI:** 10.1155/2017/8629712

**Published:** 2017-03-05

**Authors:** Piotr Hogendorf, Aleksander Skulimowski, Adam Durczyński, Anna Kumor, Grażyna Poznańska, Aleksandra Oleśna, Joanna Rut, Janusz Strzelczyk

**Affiliations:** ^1^Department of General and Transplant Surgery, Medical University of Lodz, 22 Kopcinskiego St., 90-153 Lodz, Poland; ^2^Department of Pulmonology and Allergy, Medical University of Lodz, 22 Kopcinskiego St., 90-153 Lodz, Poland; ^3^Department of Anesthesiology and Intensive Care, Medical University of Lodz, 22 Kopcinskiego St., 90-153 Lodz, Poland

## Abstract

*Background*. Proper diagnosis of pancreatic lesion etiology is a challenging clinical dilemma. Studies suggest that surgery for suspected pancreatic ductal adenocarcinoma (PDAC) reveals a benign lesion in 5% to 13% of cases. The aim of our study was to assess whether routinely used biomarkers such as CA19-9, Ca125, Ca15-3, and CEA, when combined, can potentially yield an accurate test predicting pancreatic lesion etiology.* Methods*. We retrospectively analyzed data of 326 patients who underwent a diagnostic process due to pancreatic lesions of unknown etiology.* Results*. We found statistically significant differences in mean levels of all biomarkers. In logistic regression model, we applied levels CA19-9, Ca125, and Ca15-3 as variables. Two validation methods were used, namely, random data split into training and validation groups and bootstrapping. Afterward, we built ROC curve using the model that we had created, reaching AUC = 0,801. With an optimal cut-off point, it achieved specificity of 81,2% and sensitivity of 63,10%. Our proposed model has superior diagnostic accuracy to both CA19-9 (*p* = 0,0194) and CA125 (*p* = 0,0026).* Conclusion*. We propose a test that is superior to CA19-9 in a differential diagnosis of pancreatic lesion etiology. Although our test fails to reach exceptionally high accuracy, its feasibility and cost-effectiveness make it clinically useful.

## 1. Introduction

Pancreatic ductal adenocarcinoma (PDAC) has a relatively low incidence; however, it is associated with a dismal prognosis. It is the fourth cause of cancer-related deaths worldwide, as only 5% of patients survive up to 5 years after the diagnosis [1]. Notably, mortality from pancreatic cancer has not decreased as it has for other cancers in recent years [2]. The main reasons underlying this situation are the lack of PDAC specific symptoms, its late manifestation, and high aggressiveness. Another problem that clinicians are facing is the lack of a highly effective treatment strategy for PDAC. Nowadays, the surgical treatment is the only curative option for PDAC, yet only 10–20% of patients with newly diagnosed PDAC will be suitable for pancreatic resection. Unfortunately, R0 or R1 resection does not modify patients 5-year survival rate dramatically, as it is not higher than 7–32% [3, 4].

Taking into account these issues, current research focuses on the early detection of PDAC, as not only does it increase the probability of R0 resection but also it is associated with longer survival of the patients [5]. Another clinical challenge that is widely studied is the proper diagnosis of etiology of the encountered pancreatic lesion. As there is no 100% specific and sensitive marker of PDAC and radiological signs of PDAC and inflammatory processes affecting pancreas are often misleading, misdiagnosis is relatively frequent. Studies show that surgery reveals benign lesion in a case of 5% to 13% of patients initially diagnosed with PDAC [6–11]. This situation has a great impact on patients' later quality of life as well as their further prognosis. In their randomized controlled trial comparing results of Frey procedure and pylorus preserving pancreatoduodenectomy (PD) for chronic pancreatitis, Bachmann et al. [12] reported that in 15-year follow-up the quality of life was better after Frey procedure in terms of the physical status. Moreover, patients treated with Frey procedure had longer mean survival time (14.5 ± 0.8 versus 11.3 ± 0.8 years; *p* = 0.037). Previously published trials also showed that organ sparing procedures such as Frey or Beger procedures yield better outcome than more aggressive treatment with PD [13]. Thus, not only should the ideal biomarker of PDAC be detectable on the early stage of carcinogenesis, but also it should be highly accurate as patients with benign lesions greatly benefit from organ sparing surgical techniques. Currently, there are a number of studies reporting the existence of potential biomarkers possessing such properties [14–18]. Nevertheless, as their performance is yet to be validated and they are still commercially unavailable, there is a need to fully assess the usefulness of routinely used cancer biomarkers. While CA19-9 is considered as a standard for PDAC diagnosis, its deficiency is obvious as it is not secreted by around 10% of the population because of the lack of a functional Lewis enzyme. Moreover, its high levels are present in other diseases, and its diagnostic accuracy falls significantly in jaundiced patients, to name a few drawbacks [19–21]. Nevertheless, a recent meta-analysis showed that CA19-9 has a diagnostic accuracy of 80% and remains the most useful PDAC biomarker that is routinely measured [22]. There is a growing body of evidence that other biomarkers, such as Ca125 and Ca15-3, can also play an important role in PDAC diagnosis and the prediction of its resectability or survival among patients after surgery or chemotherapy [23–29].

Considering the differential diagnosis of the pancreatic mass, some studies suggest that Ca125 complements CA19-9. Namely, their simple combination may yield a test that improves accuracy over the routinely used measurement of CA19-9 [19, 30, 31].

Taking into account these findings, our aim was to assess whether routinely used biomarkers, such as CA19-9, Ca125, Ca15-3, and CEA, when combined, can potentially yield an accurate test predicting pancreatic lesion etiology.

## 2. Material and Methods

**Figure 1:**
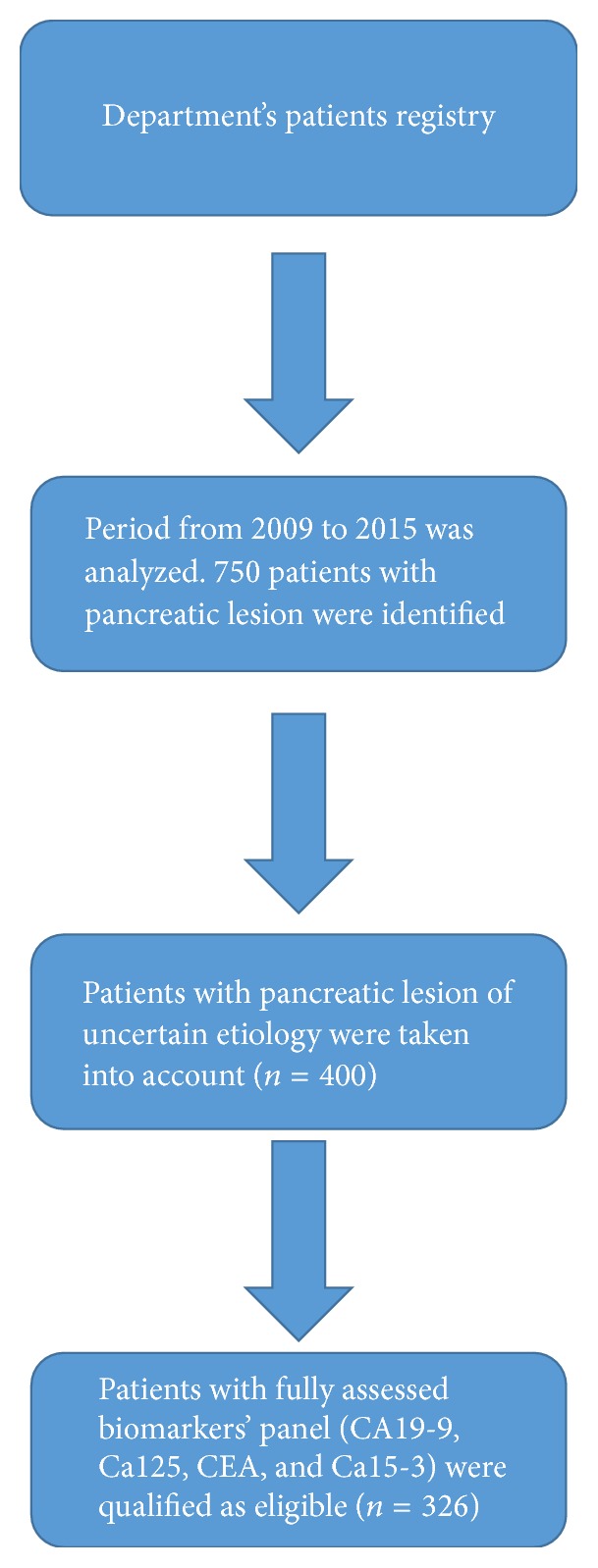


We retrospectively analyzed the Department's patients registry in order to identify the patients who underwent the diagnostic process due to pancreatic lesions of unknown etiology. From 2009 to 2015, 400 of such patients were admitted to our Department. Out of them, 326 had a panel of CA19-9, Ca125, Ca15-3, and CEA assessed on the day of the hospital admission. This group of patients was considered as eligible for our study (Figure 1). Briefly, a day before surgery, a 4,9 mL blood sample was collected (Serum Z, Sarstedt) and centrifuged for 10 min at 3000 rpm at 4°C. Serum was analyzed by the enzyme linked fluorescence assay (ELFA) using a VIDAS PC analyzer (BioMérieux). In order to divide study population into 2 subgroups, namely, patients with PDAC and patients with a benign lesion, we applied the results of pathohistological examination as a mean of the final diagnosis.

As for statistical analysis, we used Mann–Whitney nonparametrical test to compare mean biomarker levels between groups. The same test was used in order to evaluate an association between gender and the level of biomarkers, separately for a group of PDAC patients and nonmalignant patient. In the same manner, Spearman's correlation test was applied to assess the impact of age on the levels of biomarkers.

Diagnostic performance of a single biomarker was measured by building receiver operating characteristic (ROC) curves and choosing the optimal cut-off point afterward. To compare ROC curves area under the curve (AUC), we used a method developed by DeLong et al. [32].

A combined diagnostic test was created after ln-transformation of biomarkers' levels, which were used in logistic regression model later on. Other models, such as logistic regression with interaction terms, were also tested, yet it was the simple logistic regression model that yielded the best results (data not shown).

To validate our model, we randomly divided the study population into the training group (228 patients) and the validation group (98 patients). Moreover, a model created for the training group was validated using bootstrapping method (bias-corrected and accelerated method-BC_a_) [32] (*n* = 1000 bootstrap data sets). In our study, bootstrapping consisted in randomly resampling the data with replacement and repeatedly creating a logistic regression model. Such an approach ensures that obtained coefficients and 95% confidence intervals are not biased by a number of samples that radically affect data distribution of a certain biomarker.

Finally, we compared the sensitivity and specificity of the resulting model with those of CA19-9, as up to date it is the only standardly measured biomarker of PDAC that has a diagnostic and a prognostic value [33].

The statistical analysis was performed using IBM SPSS Statistics for Windows, version 20.0 (IBM Corp., Armonk, NY) and MedCalc for Windows, version 13.0 (MedCalc Software, Ostend, Belgium).

Ethical approval for this study (Ethical Committee Number RNN/367/12/KB) was provided. All patients provided written informed consent for the study.

## 3. Results

**Figure 2:**
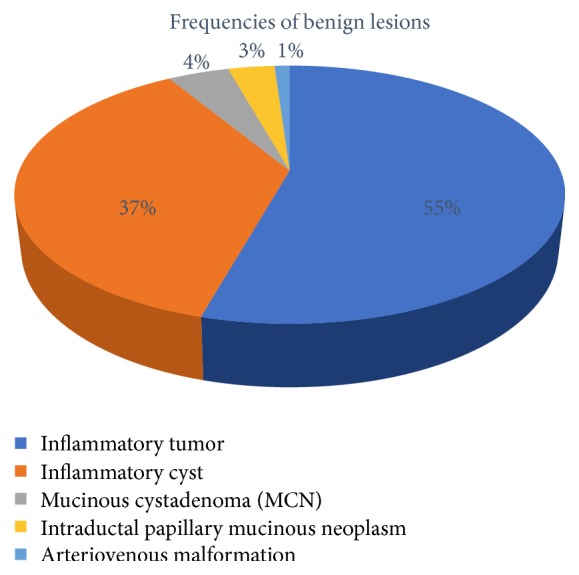


The basic demographical data concerning study population is shown in Table 1, while the frequency of various benign lesions is presented in Figure 2. Namely, 50 patients had an inflammatory tumor, and 34 had inflammatory cyst diagnosed, while 4 of them had mucinous cystadenoma of the pancreas. Three patients had intraductal papillary mucinous neoplasm and 1 was diagnosed with arteriovenous malformation.

There were no statistically significant differences associated with gender, while patients with PDAC were statistically significantly older than patients with nonmalignant lesions (*p* < 0,0001). To be exact, age >65 was associated with the presence of PDAC (HR: 1,94; 95% CI: 1,14–3,3; *p* = 0,015). Moreover, age and high levels of Ca125 and CEA and Ca15-3 moderately correlated with the presence of the unresectable lesion in PDAC group (Spearman's *ρ*_age_ = 0,165, *p* = 0,011; *ρ*_Ca15-3_ = 0,15, *p* = 0,037; *ρ*_CEA_ = 0,195, *p* = 0,006; *ρ*_Ca125_ = 0,173, *p* = 0,024).

**Figure 3:**
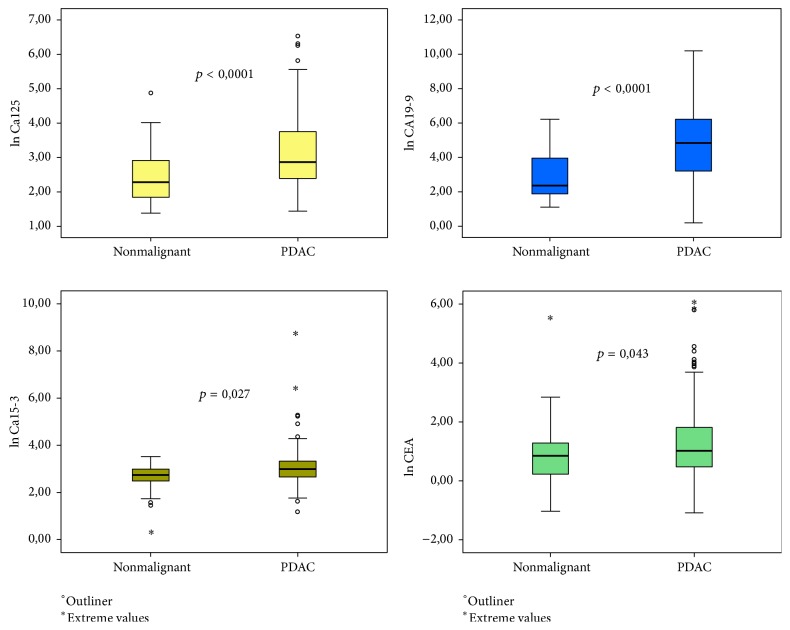


As for the distribution of biomarker levels, the box plots created with ln-transformed values (Figure 3) clearly show that no single biomarker is characterized by the distribution that would entail high performance regarding discriminating a nonmalignant lesion from PDAC. Yet, the differences between mean values of these biomarkers were statistically significant, with CA19-9 and Ca125 being the most significant ones (mean CA19-9_PDAC_ 635,05 IU/mL ± 2443,77 IU/mL versus mean CA19-9_benign_ 62,36 IU/mL ± 134,7 IU/mL, *p* < 0,0001, and mean Ca125_PDAC_ 45,37 IU/mL ± 89,04 IU/mL versus mean Ca125_benign_ 12,74 IU/mL ± 16,65 IU/mL, *p* < 0,001) (Table 2).

**Figure 4:**
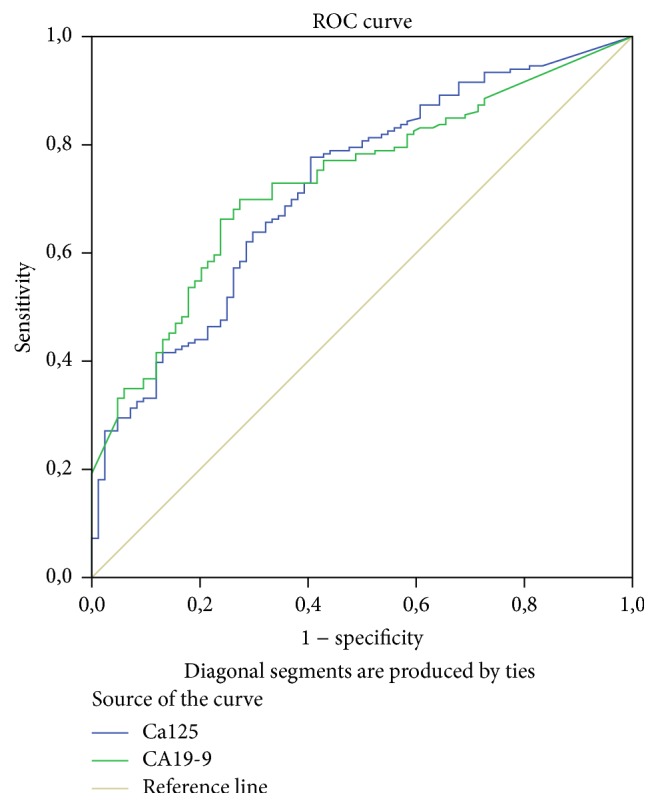


In accordance with these facts, the ROC curve for CA19-9 had the highest AUC of 0,736 followed by the ROC curve for Ca125 with AUC of 0,716. Interestingly, when comparing these curves the difference between them was not statistically significant (*p* = 0,79) (Figure 4 and Table 3). The AUC of the ROC curves for other biomarkers were not high enough to consider them as independent biomarkers for differentiating pancreatic lesion etiology.

Consequently, using ln-transformed values of biomarkers, we chose logistic regression model as an optimal method for creating a diagnostic test. After splitting the study population randomly into training and validation groups, we applied the chosen model to the training group. We also used the bootstrapping method-BC_a_ with 1000 data sets. The model summary is shown in Table 4. The coefficients in our model are as follows:* 0,253 + 1,039 ∗ CA19-9 + 1,003 ∗ CA125 + 1,048 ∗ CA15-3*. Afterward, the model was also applied to the validation group and, finally, to the whole study population. As it is shown in Table 5, the AUC of ROC curves built for these groups did not differ significantly (AUC_training_ = 0,804; AUC_validation_ = 0,791; AUC_study  group_ = 0,801).

Judging from these facts, our model is well-fitted not only because of positive cross-validation on both the training and validation group but also because of the fact that the 95% CI of coefficients calculated from logistic regression model are closely matching those calculated with the bootstrapping method. Thus, it may be assumed that the calculated model is not biased by the outliers that could highly affect our data.

**Figure 5:**
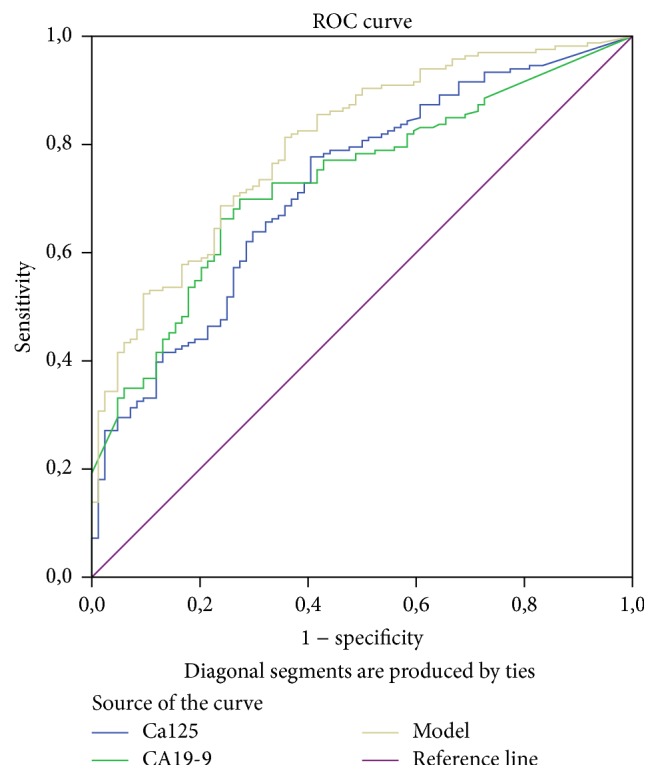


Using Youden's index, we chose an optimal cut-off point of ≥0,5 for our model, achieving the sensitivity of 81,2% and specificity of 63,10%. These values outweigh accuracy of CA19-9, which with the clinical cut-off point (≥36 IU/mL) has the sensitivity of 58,97% and specificity of 79,35%. Similarly, CA125 with optimal cut-off point ≥8,5 IU/mL is also inferior with the sensitivity of 79% and specificity of 52,17%. We analyzed this data further by comparing ROC curves (Figure 5 and Table 6). Our proposed model has superior diagnostic accuracy to both CA19-9 (*p* = 0,0194) and CA125 (*p* = 0,0026).

Observed differences in the diagnostic accuracy have clinical ramifications. When analyzing PDAC patients' group, 19,66% of patients have false negative result of the proposed model, while 41,89% of patients have false negative result of CA19-9. On the other hand, our model yields 38% of false positive results, while CA19-9 has 19,56% of false positive results. In terms of unresectable lesions these changes are not crucial, as the palliative management of these tumors, despite etiology, is quite similar. Therefore, we analyzed subgroups of patients resectable lesions, who benefit most from proper diagnosis, since they can be qualified for the adequate surgical treatment. As shown in Table 7, the proposed model has higher true positive rate in the resectable PDAC subgroup (69,7% versus 54,54%) yet it also has higher rate of false positive results in the subgroup of resectable benign lesions (22,64% versus 9,43%)

Weighting risk/benefit ratio in this setting is relatively difficult, as the proposed model outweighs the accuracy of CA19-9 in the detection of resectable PDAC, yet it creates a higher risk of qualifying patients with a benign lesion to the PD. However, taking into account the fact that PD is the only curative treatment option for PDAC and it significantly improves the survival it seems that this advantage makes our model superior to the CA19-9 alone. Moreover, it should be also underlined that in some cases of inflammatory tumors Whipple procedure is preferred because of tumor's size or/and the infiltration of the neighboring tissue. Therefore, in our opinion the improvement of the diagnostic accuracy of the early stage PDAC is a vital asset.

## 4. Discussion

Despite current advances in imaging techniques, the minimally invasive procedures, and biomarker research, proper diagnosis of pancreatic cancer is still challenging. As we still lack highly specific and sensitive biomarker of PDAC, recent development in imaging techniques seems promising. Multidetector flow CT (MDCT) and EUS are accepted as the most effective means of PDAC diagnosis. Yamada et al. reported that MDCT has 90,4% accuracy in distinguishing PDAC from benign lesion [34]. Similarly, Zhang et al. [35] showed that fusing images obtained by PET/CT and contrast-enhanced CT yields 94,3% accuracy in differentiating benign lesion from the malignant one. As for EUS, its modalities, such as contrast-enhanced EUS or EUS with digital image analysis, have similar accuracy to the techniques mentioned above in terms of lesion etiology differentiation [36–38]. Yet, it should be noted that these techniques are not widespread, and up to date and conventional EUS-FNA has much lower sensitivity and moderately lower specificity [38].

Taking into account the fact that these techniques are mainly used in highly specialized institutions that also have experienced clinicians performing these procedures, it seems that advances in PDAC biomarkers might improve this cancer diagnosis rate in institutions of the lower reference level. As symptoms of PDAC are nonspecific and its differentiation with benign lesion is challenging, the early diagnosis of cancer greatly depends on General Practitioner/family doctor and/or community hospitals capabilities.

As intricate imagining techniques are often unavailable, especially in smaller communities and less developed regions, the proper use of available biomarker tests should be highlighted, as they are cost-effective and easy to perform. It should be also noticed that the incidence of benign lesion found on histopathologic examination after pancreaticoduodenectomy is still relatively high; hence novel tools for differential diagnosis are of uttermost importance.

In our study group, patients with PDAC were statistically significantly older than patients with nonmalignant lesions (*p* < 0,0001). Moreover, age >65 and high levels of Ca125 and CEA and Ca15-3 moderately correlated with the presence of an unresectable lesion in PDAC group. These results seem to be corresponding with the incidence of PDAC which is the highest in the 7th decade of life [39, 40].

In the literature, there is no evidence of age-determined bias on PDAC cancer markers assessment. The majority of studies distinguishing chronic pancreatitis or benign pancreatic lesions from PDAC have almost the same age difference between study groups [41]. However, measurement of PDAC markers in the group of age-adjusted groups would be the most appropriate approach [42].

Currently, CA19-9 is the only recognized biomarker, a “gold standard” for PDAC diagnosis and its monitoring. Yet, it is not highly specific or sensitive, as approximately 10% of the world population do not secrete this molecule due to lack of Lewis antigen [43]. In terms of false positive results, many benign conditions, as well as other cancers, cause CA19-9 levels to increase [20, 44]. Furthermore, studies show that only around 50% of PDAC with diameter <20 mm have significantly increased CA19-9 levels [45]. Thus, Ca 19-9 levels usefulness in early PDAC diagnosis is questionable. Nevertheless, when differentiating pancreatic lesion of unknown etiology, CA19-9 has reportedly the sensitivity and specificity of 75,36% and 60,60%, respectively, which makes it a relatively useful tool [46].

Ca125 (also known as MUC16) is a protein that has a well-established role in the PDAC development, especially in the later stages; for instance, it promotes cancer cells motility and drug-resistance and reprograms PDAC metabolism [47–50]. Its clinical utility is also thoroughly assessed. As already mentioned, Ca125 alone is a relatively accurate biomarker of PDAC and its level has also impact on patients prognosis and it can indicate the resectability of a tumor. Yet, it seems that Ca125 reaches significantly high levels during the late stage of PDAC progression [23, 51, 52]. Thus, its relatively high sensitivity is hampered by low specificity.

Ca15-3 is a derivate of MUC-1, a protein that plays an essential role in PDAC development. Namely, some studies show that MUC1 promotes survival of PDAC cells by inducing hypoxia-inducible factor 1 alpha expression and modulating cells metabolism as well as promoting resistance to chemotherapeutics [53–55]. Moreover, MUC1 regulates expression of a great number of miRNAs. There is evidence that this role of MUC1 is crucial for PDAC progression, as, for instance, MUC1 influences transcription of microRNAs that are associated with the process of metastasis [56]. This data might suggest that assessment of Ca15-3 could prove a useful biomarker for PDAC diagnosis and/or patients monitoring. However, reports published up to date and our data do not confirm that Ca15-3 alone can be considered as a good biomarker of PDAC.

## 5. Conclusions

Currently, we still lack a highly accurate, easy-to-perform, and cost-effective singular biomarker of PDAC, which would enable its early diagnosis in virtually every institution. Therefore, combining the measurement of universally available biomarkers to enhance the accuracy of PDAC diagnosis seems promising. In our study, we propose a test consisting of three biomarkers that is superior to CA19-9 in a differential diagnosis of pancreatic lesion etiology. Although our test fails to reach exceptionally high accuracy, its feasibility and cost-effectiveness make it clinically useful. Given these features, it can help in determining pancreatic lesion etiology in almost every clinical setting. It might be hypothesized that its results, combined with the results from conventional imagining techniques, can enhance the rate of proper assessment of pancreatic lesion etiology.

## Figures and Tables

**Table 1:** 

	PDAC group	Benign lesions
Mean age (±SD)	62 ± 9	54 ± 13

Gender (female (%)/male (%))	89 (38,03%)/145 (61,97%)	30 (32,61%)/62 (67,39%)

Resectable (%)/unresectable (%)	66 (28,21%)/168 (71,79%)	53 (57,6%)/39 (42,4%)

Stage I/II	66	
Stage III	127	
Stage IV	41	

**Table 2:** 

		Mean (±SD) [IU/mL]	*p*	Area under curve [AUC] (95% CI)
CA19-9	PDAC	636,05 (±2443,77)	<0,0001	**0,736** (0,676–0,795)
	benign	62,36 (±134,7)		
Ca125	PDAC	45,37 (±89,05)	<0,0001	**0,717** (0,653–0,782)
	benign	12,74 (±16,65)		
Ca15-3	PDAC	46,03 (±301,33)	0,027	**0,582 **(0,515–0,649)
	benign	15,77 (±7,91)		
CEA	PDAC	11,95 (±44,5)	0,043	**0,598** (0,527–0,670)
	benign	5,44 (±25,69)		

**Table 3:** Pairwise comparison of ROC curves.

Ca125~CA19-9	
Difference between areas	0,0105
Standard error^*∗*^	0,0403
95% confidence interval	−0,0683–0,0895
*Z* statistics	0,261
Significance level	*p* = 0,7942

^*∗*^DeLong et al. 1988.

**Table 4:** 

	OR	*p*	95% CI		Bootstrap		
			Lower limit	Upper limit	*p*	95% CI	
						Lower limit	Upper limit
CA19-9	1,003	0,002	1,001	1,005	0,004	1,002	1,007
Ca125	1,039	0,009	1,010	1,070	0,02	1,016	1,084
Ca15-3	1,003	0,015	1,009	1,087	0,006	1,018	1,099
Constant	0,253	0,001	N/A	N/A	0,001	0,115	0,420

**Table 5:** 

	Area under curve (AUC)	SD	*p*	95% CI	
				Lower limit	Upper limit
Training group	0,804	0,032	<0,0001	0,741	0,867
Validation group	0,791	0,055	<0,0001	0,683	0,899
Study group	0,801	0,028	<0,0001	0,746	0,855

**Table 6:** 

	Difference between areas	SE	95% CI		*p*
			Lower limit	Upper limit	
Model versus CA19-9	0,0675	0,0289	0,0109	0,124	0,0194
Model versus Ca125	0,0780	0,0259	0,0273	0,129	0,0026
CA19-9 versus Ca125	0,0105	0,0403	−0,0684	0,0895	0,7942

**Table 7:** 

	Sensitivity	Specificity	Proper diagnosis of resectable PDAC (*n*)	Proper diagnosis of resectable benign lesion (*n*)
CA19-9	58,97%	79,35%	36/66 (54,54%)	48/53 (90,56%)
Model	81,2%	63,1%	46/66 (69,7%)	41/53 (77,36%)
	Δ = 22,23%	Δ = −16,25%	Δ = 15,16%	Δ = −13,2%
